# Efficiency of puromycin-based technologies mediated by release factors and a ribosome recycling factor

**DOI:** 10.1093/protein/gzt031

**Published:** 2013-07-03

**Authors:** Hiroyuki Ohashi, Masamichi Ishizaka, Naoya Hirai, Etsuko Miyamoto-Sato

**Affiliations:** 1 Division of Interactome Medical Sciences, The Institute of Medical Science, The University of Tokyo, 4-6-1, Shirokanedai Minato-ku, Tokyo 108-8639, Japan; 2 Department of Biosciences and Informatics, Faculty of Science and Technology, Keio University, 3-14-1, Hiyoshi, Kohoku-ku, Yokohama, Kanagawa 223-8522, Japan; 3 Department of Nano, Medical and Polymer Materials, Yeungnam University, 280, Daehak-ro, Gyeongsan, Gyeongbuk 712-749, Korea

**Keywords:** C-terminal protein labelling, *in vitro* virus, puromycin, ribosome recycling factor/release factor

## Abstract

Two puromycin-based techniques, *in vitro* virus (IVV) and C-terminal labelling of proteins, were developed based on the observation that puromycin binds the C-terminus of a protein. Puromycin technology is a useful tool for the detection of proteins and analysis of protein–protein interactions (PPIs); however, problems arise due to the existence of stop codons in the native mRNAs. Release factors (RFs) that enter the A-site of the ribosome at stop codons compete with puromycin. To overcome this issue, we have used a highly controllable reconstituted cell-free system for puromycin-based techniques, and observed efficient IVV formation and C-terminal labelling using templates possessing a stop codon. The optimal conditions of IVV formation using templates possessing a stop codon was RF (−), while that of C-terminal labelling was RF (−) and the ribosome recycling factor (RRF) (+). Thus, we have overcome the experimental limitations of conventional IVV. In addition, we discovered that RRF significantly increases the efficiency of C-terminal protein labelling, but not IVV formation.

## Introduction

**Fig. 1:**
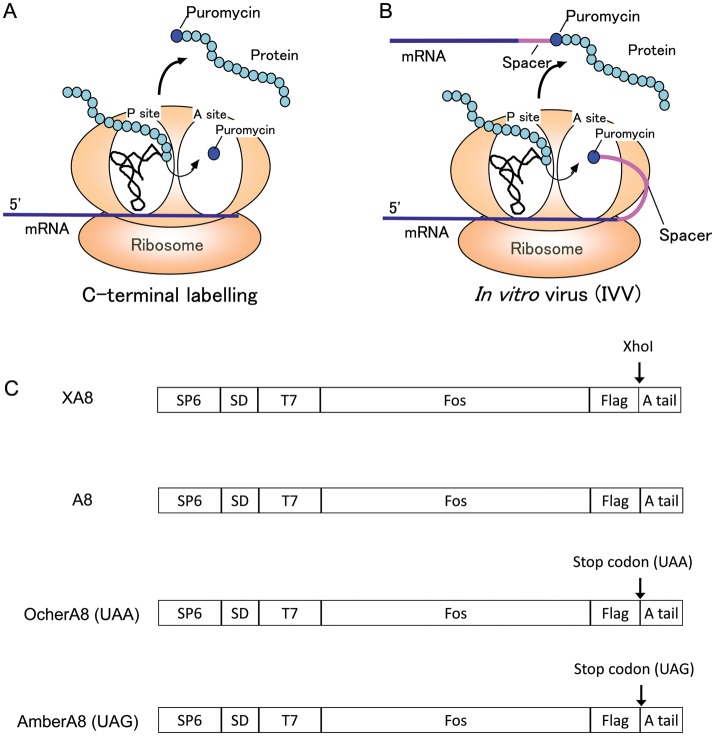
An illustration demonstrating the puromycin-based technologies. (**A**) C-terminal labelling of a protein on the ribosome. A puromycin derivative (Fluor-dCpuro) can enter the ribosomal A-site to bind covalently to the C-terminal end of the protein. (**B**) IVV formation on the ribosome. Puromycin at the 3′-terminal end of a spacer ligated to an mRNA can enter the A-site of a ribosome to covalently bind to the C-terminal end of its encoding protein. (**C**) The mRNA template for IVV formation and C-terminal labelling of proteins. The mRNA template comprises the SP6 promoter, SD sequence, T7 tag, open reading frame, Flag tag and 3′-tail. Four mRNA templates have different 3′-tail sequences: XA8 (CTCGAGAAAAAAAA), A8 (AAAAAAAA), OcherA8 (UAAAAAAAAAA) and AmberA8 (UAGAAAAAAAA).

The identification of protein–protein interaction (PPI) networks is an important aspect of proteomics research. *In vitro* selection experiments using mRNA display methods, which were originally developed for evolutionary protein engineering, such as *in vitro* virus (IVV) (Nemoto *et al.*, 1997; Miyamoto-Sato *et al.*, 2000; Miyamoto-Sato *et al.*, 2003) or mRNA–peptide fusions (Roberts and Szostak, 1997; Hammond *et al.*, 2001; Keefe and Szostak, 2001), are powerful tools for the analysis of protein function (Amstutz *et al.*, 2001, Miyamoto-Sato *et al.*, 2010). During the course of IVV development (Nemoto *et al.*, 1997), two useful puromycin-based techniques (known as ‘puromycin technology’, Fig. 1A and B) were developed based on the observation that puromycin can bind the C-terminus of a full-length protein (Miyamoto-Sato *et al.*, 2000). One of these puromycin-based techniques is IVV formation (Nemoto *et al.*, 1997), whereby an *in vitro*-translated full-length protein (phenotype) is attached to its encoding mRNA (genotype). The second puromycin-based technique is C-terminal protein labelling (*Nemoto et al., 1999*; Miyamoto-Sato *et al.*, 2000; Doi *et al.*, 2002), in which a puromycin derivative bearing a fluorescent moiety is used to label the C-terminal end of a full-length protein. Thus, high-throughput *in vitro* protein selection may be achieved by combining IVV and post-selection analysis (to confirm PPIs) using C-terminally labelled proteins, particularly during proteome exploration. The mRNA template used in IVV must satisfy one major requirement, the deletion of the stop codon. The stop codon must be deleted to prevent the release of the protein from the ribosome and stabilise IVV formation (Miyamoto-Sato *et al.*, 2010). We have previously performed IVV using a natural mRNA template (Miyamoto-Sato *et al.*, 2010; Fujimori *et al.*, 2012). Although IVV is a powerful tool for the analyses of protein–protein networks, challenges arise because of the presence of stop codons in native mRNAs. Thus, if full-length proteins or the C-terminal segments of proteins are synthesised under normal conditions, they detach from the ribosome because of the action of release factors (RFs) that enter the A-site of the ribosome and bind to the stop codons (Korostelev *et al.*, 2008; Korostelev *et al.*, 2010; Sund *et al.*, 2010; Korostelev, 2011). Such events compete with puromycin (Miyamoto-Sato *et al.*, 2000). This limitation also applies to C-terminal end labelling technology when using a fluorescent moiety. Therefore, as stop codons must be removed from the template, this technique is rendered inconvenient for conducting comprehensive proteome analyses. To overcome the limitations of current puromycin-based techniques, we have designed a RF-free *in vitro* translation system. Currently, crude cell extract-based cell-free protein synthesis systems have been the only methods suitable for *in vitro* selection. However, it is unlikely that the challenges could be overcome if a cell extract were used in the cell-free protein synthesis system, because the conventional systems contain RFs. We have previously reported that the development of a reconstituted, highly controllable cell-free protein synthesis system composed of individually prepared components required for gene expression in *Escherichia coli* is achievable (Shimizu *et al.*, 2001; Ohashi *et al.*, 2007; Ohashi *et al.*, 2010; Ohashi *et al.*, 2012;). In an earlier study, we reported a highly efficient ribosomal display using this system (Ohashi *et al.*, 2007). In the present study, we have developed a new IVV based on the reconstituted cell-free system, which is more easily controlled than conventional cell extract systems. When mRNAs containing a stop codon were translated, the translated proteins exhibited negligible IVV formation or fluorescein labelling due to RFs in the cell extract (e.g. RF1 and RF2), which inevitably compete with puromycin. However, peptide termination was overcome using a reconstituted cell-free system where RFs were absent. We investigated the effect of RFs on puromycin-based techniques for this purpose. Furthermore, we analysed the effect of ribosome recycling factor (RRF) on the formation of protein–RNA conjugates and C-terminal-labelled proteins using the reconstituted cell-free system, predicting that RRF may increase the efficiency of these reactions. Therefore, we applied the reconstituted cell-free system to the puromycin-based techniques to facilitate the comprehensive selection of genes containing termination codons, thereby overcoming the experimental limitations of conventional IVV.

## Materials and methods

### Reagents

Reagents and solvents were purchased from standard suppliers. All enzymes used in this study were from commercial sources. Two types of reconstituted prokaryotic cell-free translation systems (PURESYSTEM classic II) RF (+)/RRF (+) and RF (−)/RRF (−) with or without RFs and RRF (PURESYSTEM custom), respectively, were purchased from the Post Genome Institute Co., Ltd (Tokyo, Japan).

### Preparation of DNA

Polymerase chain reaction (PCR) amplification was conducted through 35 cycles (98°C, 20 s; 55°C, 60 s; 72°C, 180 s) with the primers listed in Table I. DNA templates of c-Fos from pCMV-FosCBPzz, including a segment of c-Fos (491–772) cloned from a mouse testis cDNA library (Takara Bio, Inc., Shiga, Japan) were amplified with the 5′-primer SP6 (O29) T7 FosFlag1A and the 3′-primer 3′ FosFlag1A. The PCR product was purified by QIAquick (Qiagen, The Netherlands) and used as a template for a second round of PCR with primers 5′-SD+T7 and 3′-FosFlag1A. The resultant PCR product was used as a template for a third round of PCR with primers 5′-uniSP6 (PURESYSTEM) and 3′-primer 3′-FosFlag1A with A8, 3′-Flag-R3 with XA8, 3′-FosFlag(s)1A with Ochre (UAA) A8 or 3′-FosFlag(s)1A2 with Amber (UAG) A8. The PCR products were then purified by QIAquick and used as templates for subsequent *in vitro* transcription.

**Table I.** Primers used in this study

Primer	Sequence
5′-SP6(O29)T7 Fos Flag 1A	GAATTTAGGTGACACTATAGAACAACAACAACAACAAACAACAACAAAATGGCTAGCATGACTGGTGGACAGCAAATG
5′-SD+T7	AAGGAGATATACCAATGGCTAGCATGACTGGTGGAC
5′-uniSP6(pure system)	GAAATATTTAGGTGACACTATAGAGACCACAACGGTTTCCCTCTAGAAATAATTTTGTTTAACTTTAAGAAGGAGATATACCA
3′-FosFlag1A	TTTTTTTTCTTGTCGTCATCGTCCTTGTAGTCCTCGAGGCCAAGGTCATCGGGGAT
3′-Flag-R3	TTTTTTTTCTCGAGCTTGTCGTCATCG
3′-FosFlag(s)1A	TTTTTTTTTTACTTGTCGTCATCGTCCTTGTAGTCCTCGAGGCCAAGGTCATCGGGGAT
3′-FosFlag(s)1A2	TTTTTTTTCTACTTGTCGTCATCGTCCTTGTAGTCCTCGAGGCCAAGGTCATCGGGGAT

### Ligation of mRNA to a Fluor-PEG Puro spacer

We prepared the Fluor-PEG Puro spacer according to the method described previously (Miyamoto-Sato *et al.*, 2003). Transcription was performed using the RiboMax Large Scale RNA Production System (Promega; Fitchburg, WI, USA) with the purified PCR products as DNA templates described in the previous section. The transcription product, mRNA (200 nM), was ligated to 40 mM Fluor-PEG Puro spacer with T4 RNA ligase (Takara) in the presence of 120 mM free polyethylene glycol (PEG) (average molecular weight 2000) at 15°C. Excess spacer was removed using RNeasy Mini Kits (Qiagen). The fluorescence of the mRNA with a Fluor-PEG spacer on the gel was directly visualised with a fluorescence image analyser (Molecular Imager FX, Bio-Rad Laboratories, Hercules, CA, USA).

### IVV formation and C-terminal protein labelling

The standard IVV formation protocol was performed using the mRNA (200 nM) ligated to a Fluor-PEG Puro spacer as a template in a reconstituted cell-free system at 37°C for 1 h. The reaction was conducted under RF (±) conditions and/or RRF (±) conditions, where RF (+) denotes 0.25 µM RF1 and 0.24 µM RF2, whereas RRF (+) denotes 0.48 µM of RRF. The translation product was analysed by 8 M urea/10% sodium dodecyl sulphate–polyacrylamide gel electrophoresis (SDS–PAGE). The fluorescence of the resulting IVV on the gel was directly visualised with a Molecular Imager FX instrument (Bio-Rad Laboratories). The Fluor-PEG Puro spacer offers an easy method for the detection of IVV without the use of any radioisotopes. The C-terminal labelling of proteins was performed using 200 nM mRNA as a template in the presence of 20 mM Fluor-dCpuro (Doi *et al.*, 2002) at 37°C for 1 h. The yield of C-terminal-labelled proteins was evaluated by scanning fluorescence emission of a 15% SDS–PAGE with a Molecular Imager FX instrument (Bio-Rad Laboratories).

## Results

**Fig. 2:**
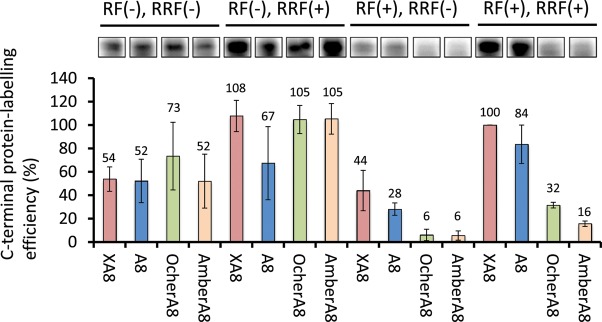
Effect of RFs, RRF and the 3′-tail of template mRNA on C-terminal protein labelling. The efficiency of C-terminally labelled proteins was examined using 200 nM c-fos mRNA containing a Flag tag followed by either an XA8, A8, OcherA8 or AmberA8 sequence. The yield of C-terminal-labelled proteins was evaluated by scanning fluorescence emission of a 15% SDS–PAGE with a Molecular Imager. The formation efficiency of C-terminally labelled proteins was normalised to the formation efficiency using XA8 mRNA under the conditions of RF (+) and RRF (+). Data represent the mean ± SD of three separate experiments.

Initially, we analysed the C-terminal labelling efficiency of proteins containing a stop codon using fluorescence to investigate the effects of RFs and RRF, and detected the dye-labelled protein synthesised using the reconstituted cell-free system. We investigated the formation efficiency of C-terminal labelled c-Fos with XA8, A8, Ochre (UAA) A8 or Amber (UAG) A8 sequences, adjacent to a FLAG tag (Fig. 1C). The A8 sequence at the 3′-end is suitable for IVV formation, and the XA8 sequence is suitable for C-terminal protein labelling (Miyamoto-Sato *et al.*, 2003). As expected, the formation efficiency of C-terminal-labelled proteins containing a stop codon (UAA or UAG) at the 3′-tail was low under RF (+) conditions, but was 3.3- to 12.2-folds higher in the absence of RFs (Fig. 2). In contrast, the templates XA8 and A8 with no stop codon were only slightly affected (∼1.9-folds higher) by the RF conditions. In addition, as expected, RRF enhanced the formation efficiency of almost all of the C-terminal-labelled templates under investigation. The optimal conditions for templates possessing a stop codon were RF (−) and RRF (+).

**Fig. 3:**
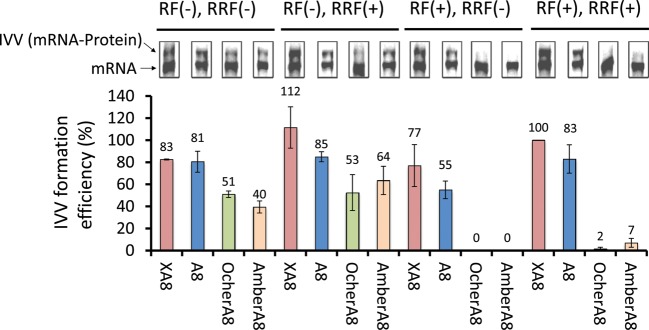
The effect of RFs, RRF and the 3′-tail of the template mRNA on IVV formation. The IVV formation was examined using 200 nM c-fos mRNA with a Flag tag followed by either an XA8, A8, OcherA8 or AmberA8 sequence. The IVV formation was analysed with 8 M urea/10% SDS–PAGE followed by staining with ethidium bromide. The electrophoretic mobility of the IVV was slower than that of the mRNA. The efficiency was normalised to the IVV formation efficiency using XA8 mRNA under the conditions of RF (+) and RRF (+). Data represent the mean ± SD of three separate experiments.

Second, we analysed IVV (mRNA–protein) formation efficiency by the detection of fluorescently labelled IVV synthesised in the reconstituted cell-free system. We investigated the effect of the 3′-tail sequences on IVV formation under RF (±) and RRF (±) conditions. The IVV products of all c-Fos mRNA templates possessed the SP6 sequence and a Shine–Dalgarno (SD) sequence in the 5′-UTR; XA8, A8, Ochre (UAA) A8 or Amber (UAG) A8 sequences were situated in the 3′-tails (Fig. 1C). The electrophoretic mobility of the IVV (Fig. 3) was slower than that of the mRNA.

The XA8 and A8 sequences in the 3′-tail were efficient (63–109%) in IVV formation under all conditions (Fig. 3). In contrast, Ochre (UAA) A8 or Amber (UAG) A8 sequences in the 3′-tail under RF (+) condition were not efficient (0–3%) in IVV formation but were rescued under the RF (−) conditions. Finally, RRF did not have a strong effect on the efficiency of IVV formation.

## Discussion

When a termination codon was present at the 3′-end for C-terminal labelling, we observed a small amount of C-terminal-labelled protein synthesis, even under RF (+) conditions, at a lower efficiency when compared with RF (−) conditions (Fig. 2). By contrast, IVV formation could barely be detected with templates possessing a termination codon under RF (+) conditions (Fig. 3). It is likely that the free puromycin (Fluor-dCpuro) can easily enter the A-site of the ribosome when compared with the puromycin linker (Fluor-PEG Puro spacer) at the 3′-end of an mRNA molecule. To confirm this hypothesis, we added 20 mM Fluor-dCpuro competitively during IVV formation using the PURESYSTEM. No IVV formation was observed in the presence of Fluor-dCpuro (data not shown). This observation suggests that 20 mM of free puromycin competes with the puromycin linker at the 3′-end of the RNA template.

Furthermore, RRF increased the efficiency of C-terminal protein labelling (Fig. 3). Presumably, the recycling of the mRNA templates after the completion of protein synthesis could account for the differences between the results of IVV formation and C-terminal protein-labelling efficiency. This mRNA recycling may provide opportunities to the free dye Fluor-dCpuro to label the C-terminus. The optimal conditions for C-terminal protein labelling with Fluor-dCpuro using mRNA templates possessing a stop codon was RF (−) and RRF (+).

When a termination codon was present at the 3′-tail for IVV formation, IVV was barely formed under RF (+) conditions, whereas a considerable amount of IVV was formed when the templates did not possess a termination codon (Fig. 2). This observation corresponds to the results from our previous study examining puromycin incorporation in a rabbit reticulocyte lysate translation system that contained RFs (Nemoto *et al.*, 1997). In that study, we observed a higher efficiency of puromycin incorporation at the C-terminus of a protein using a template possessing no stop codon when compared with a template possessing a stop codon. We presume that RFs compete with puromycin at the site of stop codons (Miyamoto-Sato *et al.*, 2000). Matsuura *et al.* (2007) observed the effect of RFs on ribosome display. Surprisingly, the formation of ternary complexes (mRNA–ribosome–protein) was identical, regardless of the presence of RFs or the RNA sequence at the 3′-end, including those with a termination codon (Matsuura *et al.*, 2007). Therefore, this approach may be particularly suitable for mRNA display methods. Moreover, while RRF did not affect the formation of IVV, it did enhance the efficiency of C-terminal labelling (Fig. 2). The role of RRF is to recycle ribosomes after the completion of protein synthesis (Janosi *et al.*, 1994). We surmise that recycling of mRNA templates after the completion of protein synthesis could cause the observed difference between the results of IVV formation and C-terminal protein-labelling efficiency. Recycling of mRNAs could also provide opportunities for the free dye Fluor-dCpuro to label the C-terminal end of proteins. In contrast, RRF action is likely to be inhibited by the puromycin spacer ligated at the 3′-end of template mRNAs. It is likely that RRF cannot enter the A-site of the ribosome because of steric hindrance by the puromycin spacer.

We have observed the rescue of IVVs containing RNA templates possessing a stop codon by using a reconstituted cell-free system without RFs. Thus, we have overcome the experimental limitation of conventional IVV.

## Conclusion

The identification of PPI networks is an important aspect of proteomics research. More specifically, IVV is a powerful tool for the identification of PPI networks. However, the application of this remarkable technique using native mRNAs is hampered by the existence of stop codons in native mRNA and the presence of RFs in conventional cell-free systems. The present study provided several advancements in bypassing these limitations. We applied a highly controllable reconstituted cell-free system to puromycin-based techniques and observed the efficient formation of IVV and C-terminal labelling using templates possessing a termination codon. We also discovered that RRF increases the efficiency of C-terminal protein labelling, but not of IVV formation. This approach overcomes the experimental limitations of conventional IVV. Thus, we believe that our results support the practical use of puromycin-based techniques for evolutionary protein engineering and comprehensive proteome research.

## Funding

This research was partially supported by Yokohama Life Sciences Institute. Funding to pay the Open Access publication charges for this article was provided by Grant-in-Aid for Challenging Exploratory Research.
